# NADPH Phagocyte Oxidase Knockout Mice Control *Trypanosoma cruzi* Proliferation, but Develop Circulatory Collapse and Succumb to Infection

**DOI:** 10.1371/journal.pntd.0001492

**Published:** 2012-02-14

**Authors:** Helton C. Santiago, Claudia Z. Gonzalez Lombana, Juan P. Macedo, Lara Utsch, Wagner L. Tafuri, Maria José Campagnole-Santos, Rosana O. Alves, José C. F. Alves-Filho, Alvaro J. Romanha, Fernando Queiroz Cunha, Mauro M. Teixeira, Rafael Radi, Leda Q. Vieira

**Affiliations:** 1 Departamento de Bioquímica e Imunologia, Instituto de Ciências Biológicas, Universidade Federal de Minas Gerais, Belo Horizonte, Minas Gerais, Brazil; 2 Departamento de Patologia Geral, Instituto de Ciências Biológicas, Universidade Federal de Minas Gerais, Belo Horizonte, Minas Gerais, Brazil; 3 Departamento de Fisiologia e Biofísica, Instituto de Ciências Biológicas, Universidade Federal de Minas Gerais, Belo Horizonte, Minas Gerais, Brazil; 4 Centro de Pesquisas René Rachou, Fiocruz, Belo Horizonte, Minas Gerais, Brazil; 5 Departmento de Bioquímica e Imunologia, Faculdade de Medicina de Ribeirão Preto, Universidade de São Paulo, Ribeirão Preto, Brazil; 6 Departamento de Bioquímica, Universidad de la República, Montevideo, Uruguay; 7 Núcleo de Pesquisas em Ciências Biológicas, Universidade Federal de Ouro Preto, Ouro Preto, Minas Gerais, Brazil; 8 Center for Free Radical and Biomedical Research, Universidad de la República, Montevideo, Uruguay; Yale School of Public Health, United States of America

## Abstract

^•^NO is considered to be a key macrophage-derived cytotoxic effector during *Trypanosoma cruzi* infection. On the other hand, the microbicidal properties of reactive oxygen species (ROS) are well recognized, but little importance has been attributed to them during *in vivo* infection with *T. cruzi.* In order to investigate the role of ROS in *T. cruzi* infection, mice deficient in NADPH phagocyte oxidase (gp91*^phox^* ^−/−^ or *phox* KO) were infected with Y strain of *T. cruzi* and the course of infection was followed. *phox* KO mice had similar parasitemia, similar tissue parasitism and similar levels of IFN-γ and TNF in serum and spleen cell culture supernatants, when compared to wild-type controls. However, all *phox* KO mice succumbed to infection between day 15 and 21 after inoculation with the parasite, while 60% of wild-type mice were alive 50 days after infection. Further investigation demonstrated increased serum levels of nitrite and nitrate (NOx) at day 15 of infection in *phox* KO animals, associated with a drop in blood pressure. Treatment with a NOS2 inhibitor corrected the blood pressure, implicating NOS2 in this phenomenon. We postulate that superoxide reacts with ^•^NO in vivo, preventing blood pressure drops in wild type mice. Hence, whilst superoxide from phagocytes did not play a critical role in parasite control in the *phox* KO animals, its production would have an important protective effect against blood pressure decline during infection with *T. cruzi*.

## Author Summary

When pathogens enter their hosts, they are fought by several resistance strategies, including capture by phagocytes and the production of pathogen-toxic molecules. Nitric oxide, a free radical, has been extensively studied as one of these toxic molecules that successfully mediates intracellular parasite killing, including *Trypanosoma cruzi*, the protozoan parasite that causes Chagas' disease. On the other hand, reactive oxygen species also mediate resistance to several pathogens, mainly bacterial. In this study, we addressed the role of reactive oxygen species in the resistance to *T. cruzi* using gene-deficient mice, a species which phagocytes lack the ability to produce (*phox−/−* mice). We found that phagocyte-derived reactive oxygen species are not critical to mediate resistance to parasite in the knock-out animals. However, *phox−/−* mice presented higher mortality and lower blood pressure due to infection with *T. cruzi* than non-deficient mice. The blood pressure was restored to normal by an inhibitor of nitric oxide synthesis by phagocytes. We hypothesize that superoxide (one of the oxygen reactive species) controls blood pressure during infection with *T. cruzi,* by reacting with nitric oxide and preventing its action on blood vessels.

## Introduction

For a long time, reactive oxygen species (ROS) were considered the main anti-microbial radical produced by the immune system, playing a role against bacterial, fungal and protozoa infections. After the discovery of nitric oxide (^•^NO), ^•^NO found to play a major role in host defense, especially against protozoan parasites. A role against *Toxoplasma* [1], [2], *Plasmodium* [3] and *Leishmania* [4], [5] infections was still attributed to ROS, albeit in some cases this role remains a matter of debate [6], [7], [8], [9].

Since ^•^NO was found to be one of the most important IFN-γ-induced anti-parasitic mechanisms, the studies about its role in different diseases was intensified. The advent of gene knockout (KO) technology allowed the dissection of the real extent of ^•^NO involvement in parasitic diseases. ^•^NO was found to be crucially important in a variety of infections [10], [11], however, NOS2-deficient animals are less susceptible than *ifn-γ* KO to most microorganisms studied [12], [13], [14], [15], [16]. So, the search for other mechanisms of host resistance induced by IFN-γ started, and the interest in ROS warmed up again.

*Trypanosoma cruzi* is an intracellular parasite associated with high morbidity during both acute and chronic phases of infection. Resistance to this parasite is **mostly** driven by IFN-γ. This cytokine mediates the control of parasite proliferation in tissues and blood in a NOS2-dependent way. However, ^•^NO may not be necessary for host resistance to *T. cruzi* infection when less virulent strains are used [13]. In addition, previously published data suggest that NOS2 deficient mice exhibit delayed mortality when compared to *ifn*-γ KO mice [13], [14], denoting an additional effector mechanism involved in *T. cruzi* immune resistance. Further studies suggested IFN-γ-induced p47GTPase LRG-47 as one major factor of resistance to *T. cruzi* infection along with ^•^NO [17], [18]. Although there is convincing evidence for the effects of ROS-induced damage to *T. cruzi in vitro* [19], [20], the role of these reactive species *in vivo* has not yet been addressed.

*In vitro*, *T. cruzi* is readily phagocyted by macrophages and triggers respiratory burst [19], [21]. However, production of ROS alone is not sufficient to kill parasites inside these cells [20], [21], and activation by IFN-γ, induction of NOS2 and production of ^•^NO are required [20], [21], [22]. In the infected macrophage, ^•^NO reacts with superoxide yielding peroxynitrite [21], which is a powerful oxidant and seems to be the main effector molecule against *T. cruzi* [19]. Peroxynitrite is more efficient to kill *T. cruzi* epimastigotes *in vitro* than superoxide or ^•^NO alone [19]. Moreover, evidence of peroxynitrite production during *in vitro* and *in vivo* infection with *T. cruzi* is available, as nitrated proteins are found both in macrophages and in mouse and human tissues [23], [24]. Indeed, it has just been reported that internalized trypomastigotes in activated macrophages are killed by peroxynitrite-dependent mechanisms [21]. The importance of nitro-oxidative mechanisms is underscored by the finding that virulent *T. cruzi* strains, which naturally have high peroxiredoxin levels [25], and strains overexpressing peroxiredoxins [21], [26] are protected from peroxynitrite and macrophage-dependent nitro-oxidative killing (peroxiredoxins readily decompose peroxynitrite). Albeit nitration of proteins *in vivo* may be achieved independently of peroxynitrite, it is still dependent on the production of superoxide and ^•^NO [23], [24], [27] Hence, parasite damage is dependent not only on ·NO, but on both superoxide and nitric oxide.

In order to investigate the contribution of ROS in resistance to *T. cruzi* infection, mice deficient in the gp91*^phox^* (*phox* KO) subunit of NADPH oxidase, a model for chronic granulomatous disease [28], were used. These animals fail to produce ROS in endothelial cells, causing a defect in endothelium-derived relaxation of arteries [29], [30], and in phagocytic cells, leading to deficient resolution of bacterial and fungal infections [28]. Although these animals were found somewhat more susceptible to *Leishmania donovani* [5], their susceptibility to *L. major* is still a matter of debate [4], [6]. In the present study, *phox* KO mice were found to succumb to infection with *T. cruzi*, despite adequate control of parasite replication. The immunological and physiological functions of ROS in such model were investigated.

## Methods

### Ethics statement

The procedures used in this study were approved by the Animal Ethics comittee at the Universidade Federal de Minas Gerais, protocol number 031/09. All care was taken to minimize animal suferring.

### Animals

Inbred C57BL/6 (WT) mice (males and females, 4–6 week old) were used as controls (CEBIO, Instituto de Ciências Biológicas, UFMG, Belo Horizonte, MG, Brazil). Animals were kept in a conventional animal facility at controlled temperature, light/dark cycles and environmental barriers. The gp91*^phox^*-deficient (*phox* KO) [28] and IFN-γ- deficient (*inf-γ* KO) [31] mice, both in C57BL/6 background, were purchased from The Jackson Laboratories (Bar Harbor, ME, USA) and bred under specific pathogen free conditions at the Gnotobiology Laboratory, Departmento de Bioquímica e Imunologia, ICB, UFMG.

### Parasite, infection, cytokines and serum NOx measurements

*T. cruzi* (Y strain) was maintained by weekly passage in Swiss mice. For in vivo experimental infections, mice were inoculated i.p. with 1000 blood-stage trypomastigotes. The parasitemia was evaluated by counting parasites in 5 µL of blood drawn from the tail vein [32]. Mortality of infected mice was monitored daily. Spleen cell cultures were performed as previously described [32]. Briefly, splenocytes from infected mice were obtained on day 10 after infection, and cultured at 5×10^6^ cells/ml, in 24-well plates, with RPMI 1640 supplemented with 10% FCS, 2 mM l-glutamine, 0.05 mM 2-mercapto-ethanol, 100 U/ml penicillin, and 100 µg/ml streptomycin. Cultures were maintained at 37°C in 5% CO_2_ atmosphere. Supernatants were harvested 72 hours later for TNF and IFN-γ measurements. Mice were bled on days 0, 10 and 15 after infection and the level of serum cytokines was evaluated. IFN-γ and TNF were measured as described previously using specific ELISA kits (R&D Systems, Minneapolis, MN, USA) following the manufacturer's protocol. Nitrate was reduced to nitrite in lipid-free serum with nitrate reductase and measured by the Griess colorimetric reaction [33]. ELISA and immunohistochemistry for 3-nitrotyrosine (or nitrated proteins) was performed as previously described [24].

### Quantification of parasite tissue loads and *nos2* mRNA expression by real-time PCR or real-time RT-PCR

Real-time PCR for parasite quantification was performed as described previously [34] with minor modifications. Briefly, on different days after infection, heart, spleen, and liver were digested with proteinase K, followed by a phenol-chloroform-isoamyl alcohol affinity extraction. Real-time PCR using 50 ng of total DNA was performed on an ABI PRISM 7900 sequence detection system (Applied Biosystems) using SYBR Green PCR Master Mix, according to the manufacturer's recommendations. The equivalence of host DNA in the samples was confirmed by measurement of genomic IL-12p40 PCR product levels in the same samples. Purified *T. cruzi* DNA (American Type Culture Collection) was sequentially diluted for curve generation in aqueous solution containing equivalent amounts of DNA from uninfected mouse tissues. The following primers were used for *T. cruzi* genomic DNA, TCZ, GCTCTTGCCCACACGGGTGC (forward), and CCAAGCAGCGGATAGTTCAGG (reverse); and for genomic *il-12p40*, GTAGAGGTGGACTGGACTCC (forward) and CAGATGTGAGTGGCTCAGAG (reverse).

Total RNA was isolated from spleens of WT and *phox* KO infected or non-infected mice and real-time RT-PCR was performed on an ABI PRISM 7900 sequence detection system (Applied Biosystems) using SYBR Green PCR Master Mix (Applied Biosystems) after RT of 1 µg RNA using SuperScript II reverse transcriptase (Invitrogen Life Technologies). The relative level of gene expression was determined by the comparative threshold cycle method as described by the manufacturer, whereby data for each sample were normalized to hypoxanthine phosphoribosyl transferase and expressed as a fold change compared with uninfected controls. The following primer pairs were used: for hypoxanthine phosphoribosyl transferase, GTTGGTTACAGGCCAGACTTTGTTG (forward) and GAGGGTAGGCTGGCCTATAGGCT (reverse); *nos2*, CAGCTGGGCTGTACAAACCTT (forward) and CATTGGAAGTGAAGCGTTTCG (reverse).

### Hepatic and pancreatic function

Serum AST and serum Amylase were measured in sera of infected and control animals using commercially available kits and following manufactures instructions (KATAL, Belo Horizonte, MG, Brazil).

### Determination of blood pressure by tail-cuff

After exposed for 5 minutes to a white lamp, WT and *phox* KO mice were placed in a plastic restrainer. Tail blood pressure (TBP) from the animals was measured using a pneumatic cuff placed in the base of the tail with a distally attached pulse sensor. Mice were allowed to adjust to this procedure three times a week for two weeks before experiments were performed. TBP values were recorded on a tail-cuff plethysmography Model MK-2000 using Windaq software to analyze the data. At least 10 good measurements for each animal were obtained per time point and the average of selected 5 bests readings were used as TPB for an animal (n = 6 animals per group).

### Determination of blood pressures by carotid catheterization

Mean arterial pressure (MAP) was recorded continuously in anesthetized animals by Biopac System (model MP150 A-CE, Biopac Systems, CA, USA) like described previously. In brief, mice were anesthetized by using urethane (1.2 g/kpv) administered by intraperitoneal injection at different points after infection with *T. cruzi*. The adequacy of anesthesia was verified by the absence of a withdrawal response to nociceptive stimulation of a hindpaw. The left common carotid artery was exposed through a 1.0- to 1.5-cm midline incision in the ventral neck region. A catheter from polyethylene tubing (PE 5 Intramedic, Clay Adams, Becton Dickinson, Franklin Lakes, NJ, USA) was inserted approximately 0.25 cm into the common carotid artery and connected to pressure transducers. Supplemental doses of urethane (0.1 g/kg IV) were administered if necessary. The data were converted from digital to numeric form using acquisition software. Data were processed by calculation of 10-min means of MAP variable. Results are expressed as means ± SE. (measured in millimeters of mercury) of 2–6 animals per time point pooled from 3 independent experiments.

### Treatment with iNOS inhibitors

Animals were treated with 1400SW, a NOS2 inhibitor (15 mg/Kg), i.p. on days 15 and 16 after infection with *T. cruzi*. On day 16, 1400W was administered 1 h before measuring MAP. During survival experiments, 1400W (20 mg/Kg) was administered i.p. daily divided in two doses or once a day beginning on day 13 after infection (a time found to not affect parasite control with NOS2 inhibition [35] and before MAP starts declining) for 10 days. Mice treated with vehicle were used as controls. Alternatively, animals were treated with aminoguanidine (1% w/v) in drinking water from day 13 after infection.

### Statistics

The significance of differences between sample means was determined by Student's *t* test to compare WT to *phox* KO group or one-way ANOVA if *inf* KO animals were being compared as well. A mortality difference was tested using Mantel-Cox test and groups compared using one-way ANOVA. A value of p<0.05 was considered significant.

## Results

**Figure 1:**
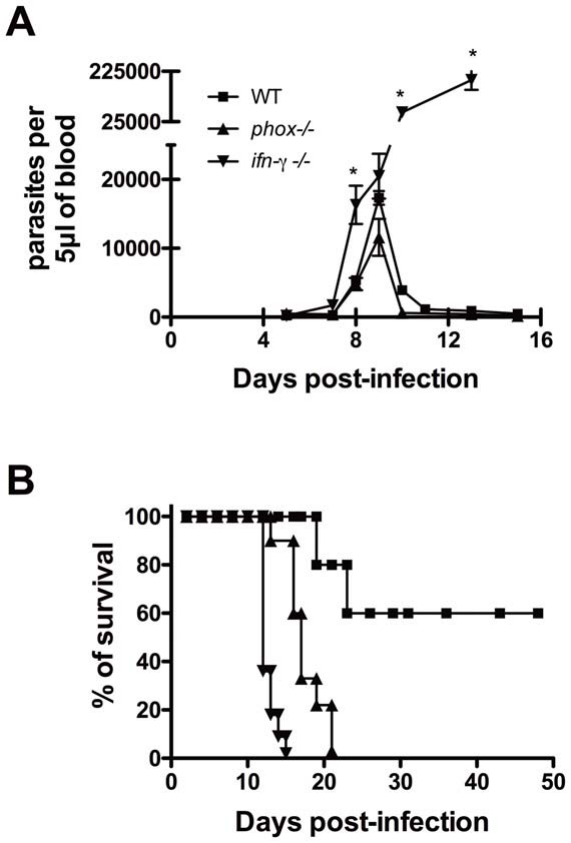
NADPH oxidase deficient-mice control parasitemia, but succumb to infection with *T. cruzi*. WT, *phox* KO and *inf-γ* KO mice were infected with 1000 blood-born trypomastigotes of Y strain of *T. cruzi*. Parasitemia (A) and mortality (B) were accessed daily. (A) Points represent mean ± SE of 5 animals per group of one from three different experiments performed with similar results. Asterisks represent P<0.05 by Student's *t* test. (B) Mortality curve is pooled from three different experiments and P<0.05 among all groups in the graph.

**Figure 2:**
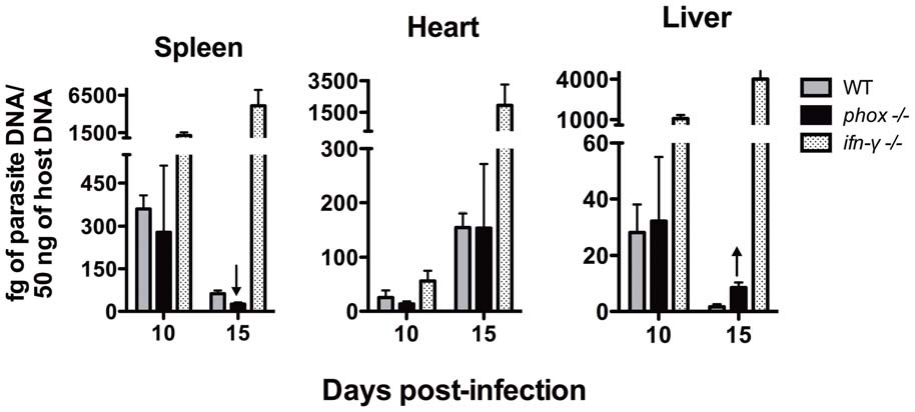
*phox* KO mice control parasite proliferation in target organs. WT, *phox* KO and *inf-γ* KO mice infected with *T. cruzi* were sacrificed on days 10 and 15 post-infection and tissue parasitism in spleen, heart and liver evaluated by real-time PCR as described in material and methods. Bars represent mean ± SE of four animals per group. Arrows indicate P<0.05 between WT and *phox* KO animals. The parasitism of *ifn-γ* KO group is statistically different from WT and *phox* groups in all organs and times analyzed, except for the heart at day 10 post-infection.

Mice deficient in functional NADPH oxidase control *T. cruzi* proliferation, but do not survive infection. *T. cruzi* infection is known to induce a strong oxidative stress in the host with high production of ROS and NO leading to nitration of serum and target organs proteins [24]. Results from our lab have shown that not only the ROS production is deficient in gp91*^phox^* NADPH oxidase (*phox* KO) genetically deficient mice as described before [28], but the level of nitration of serum proteins induced by *T. cruzi* infection in *phox* KO mice is only 25% of that observed in WT controls (data not shown). To better investigate the role of ROS in *T. cruzi* infection in vivo, *phox* KO animals were inoculated with the Y strain of *T. cruzi*. Because this is a reticulotropic strain, it is more appropriate to evaluate the effects of ROS deficiency in phagocytes in vivo. WT and *phox* KO mice displayed similar parasitemia, which peaked around 9 days post-infection (Fig. 1A) and was subsequently controlled. In contrast, *ifn-γ* KO mice presented uncontrolled parasite counts throughout the infection. WT mice presented 60–70% of survival after day 50 of infection and all IFN-γ-deficient mice died by day 15 of infection. Surprisingly, *phox* KO animals exhibited high mortality when compared to WT controls, starting at day 15 and reaching 100% mortality by 21 days of infection (Fig. 1B). This unexpected result led us to investigate a possible parasite proliferation in tissues. Coherently with the parasitemia data, tissue parasitism was controlled by *phox* KO and WT groups at 15 days post-infection in spleens, livers, and heart; *ifn-γ* KO animals exhibited high parasite proliferation in these organs (Fig. 2).

### *phox* KO and WT mice presented similar immune responses and pathology

**Figure 3:**
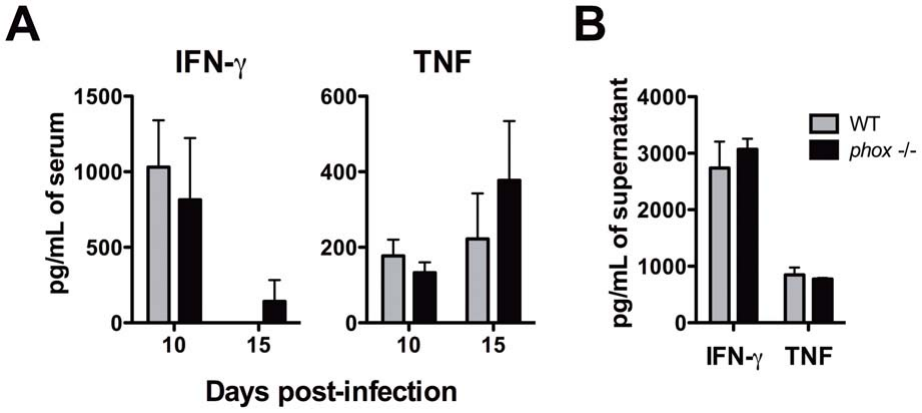
WT and *phox* KO mice produce similar levels of IFN-γ and TNF. (A) WT and *phox* KO animals infected with *T. cruzi* were bled at days 10 and 15 post-infection for cytokine measurements. (B) Infected mice were sacrificed at 10 days post-infection and spleen cells isolated and cultured for 72 hours, when supernatants were harvested. IFN-γ and TNF were measured by ELISA as described in material and methods. Bars represent mean ± SE of at least 4 animals per group. Experiment was repeated once with similar results.

The immune response from both WT and *phox* KO groups was analyzed. Both mouse strains displayed similar levels of TNF and IFN-γ in sera at 9 and 15 days post-infection (Fig. 3A). In addition, splenocytes from both groups produced expressive and equivalent levels of IFN-γ and TNF after 9 days of infection (Fig. 3B). Importantly, tissues from both animals exhibited similar quantitative and qualitative cellular infiltration in spleens, livers and hearts (not shown). Hepatic and pancreatic proofs were slightly increased after infection, but similar in both groups (Table 1).

**Table 1:** Serum AST and amylase in WT and *phox* KO mice infected with *T. cruzi.*

	*Days of infection:*	*0*	*8*	*12*	*15*
**AST** a	**WT**	60.7±10.6	219.9±39.1	139.7±34.0	119.9±78.5
	***Phox*** ** KO**	73.0±5.0	207.6±35.9	225.5±22.5	136.3±87.0
**Amylase** b	**WT**	259.2±83.4	506.2±85.1	506.9±73.5	507.4±91.8
	***Phox*** ** KO**	246.4±113.3	520.6±161.9	526.1±47.9	486.6±146.2

Values from AST and amylase are combined from 3 independent experiments with n = 3 for each independent experiment.

a AST values are expressed in IU/L;

b Amylase values are expressed in U/L.

### NOx levels were exacerbated in *phox* KO mice with possible involvement in hemodynamic disturbances

**Figure 4:**
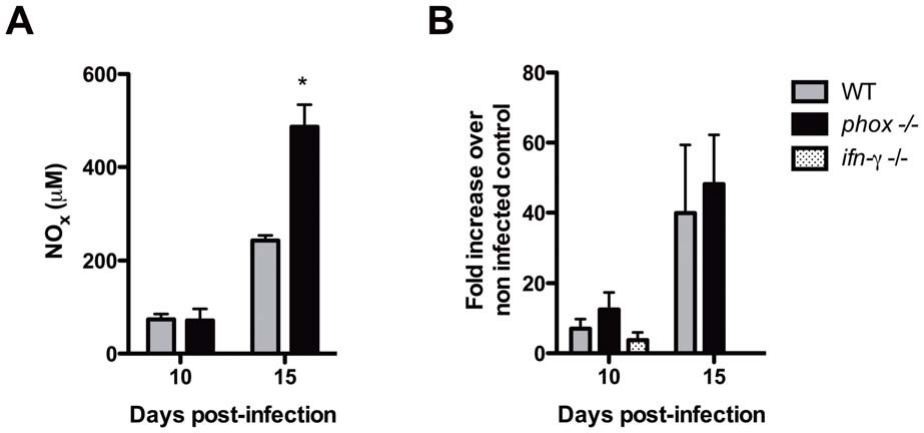
Augmented NOx levels in *phox* KO mice infected with *T. cruzi*, when compared to WT. (A) *T. cruzi*-infected mice were bled at 10 and 15 days post-infection and levels of nitrate and nitrite evaluated. Bars represent mean ± SE of 4 animals per group. Asterisks indicate P<0.05 by Student's *t* test. (B) Spleens from infected animals were harvested at 10 and 15 days post-infection and used for RNA extraction and real-time RT-PCR as described in material and methods. NOS2 expression was evaluated after normalization with HPRT constitutive gene.

**Figure 5:**
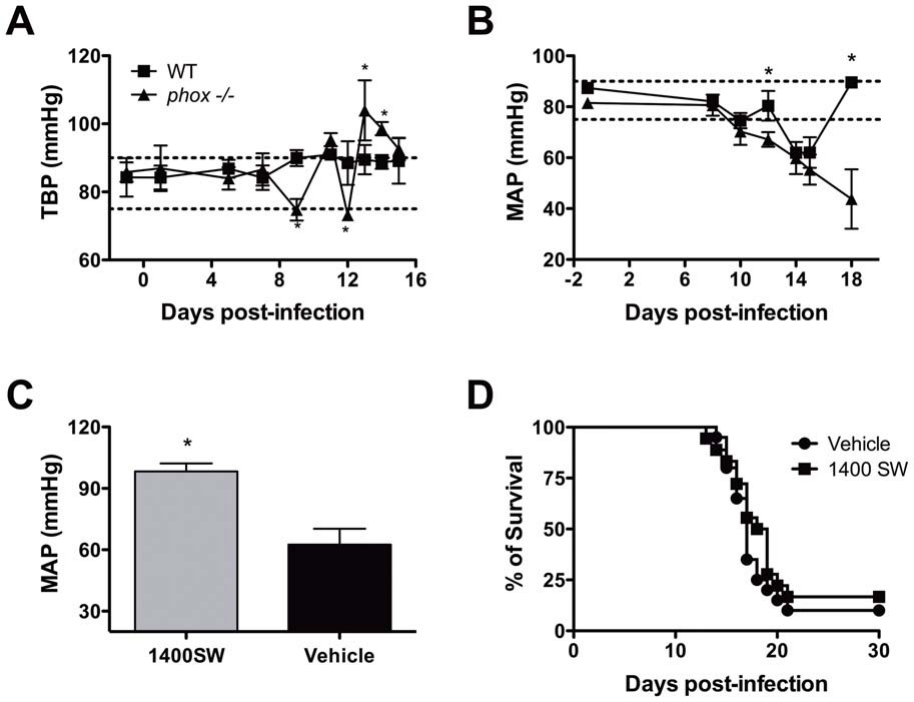
*T. cruzi-* infected phox KO mice display dramatic blood pressure variations. WT and *phox* KO animals were infected with *T. cruzi* and blood pressure evaluated in the tail (TBP) (A) or in the carotid artery (MAP) (B). Values represent mean ± SE of 6 mice of one from two performed (A) or 2–6 mice per time point pooled from 3 independent experiments (B). Asterisks represent P<0.05. (C) Drop in blood pressure is reverted by iNOS-specific inhibitor 1400SW. *phox* KO animals were infected with *T. cruzi* and blood pressure evaluated in the carotid artery (MAP) 16 days post-infection. Mice treated with 1400W received 15 mg/kg 24 and 1 hour before measurements. Values represent mean ± SE of 4 mice of one from two performed. Asterisks represent P<0.05. (D) 1400W did not revert mortality in *phox* KO mice. Mice were treated with 20 mg/kg of 1400W ip daily in single dose or divided in 2 doses starting on day 13 post-infection for 14 days. Mortality was accessed daily. Points represent mean cumulative mortality of 18–20 animals per group. Pool from 3 experiments performed with similar results (two experiments using one dose per day regime and one using two doses per day regime).

Nitrate and nitrite (NOx) levels were evaluated in serum of infected mice. *Phox* KO mice exhibited about two fold higher levels when compared to WT-infected controls (Fig. 4A). Of note, NOx levels were increased in the *phox* KO group at the same time that mice began to die, about 15 days post-infection. The expression of *nos2* gene in the liver was measured by real-time RT-PCR and both WT and *phox* KO mice displayed similar levels of mRNA (Fig. 4B). Because NOx levels closely relate with pressoric regulation, the blood pressure was evaluated in the tail (TBP) using the non-invasive tail-cuff method and in the carotid artery by catheterization, at different time points (Fig. 5). When we evaluated the blood pressure in the tail, we observed that WT mice presented a good control of pressure variation as infection progressed, but *phox* KO mice exhibited dramatic oscillations of TBP after peak parasitemia (Fig. 5A). In order to have a more accurate picture of this phenomenon, we investigated the mean arterial pressure (MAP) in a central vessel, the carotid artery. As can be observed in figure 5B, the MAP of *phox* KO mice dropped from levels between 80–90 mmHg before infection to 70–60 mmHg by the time the NOx levels starts to increase in the serum, at day 8 post-infection, and further down as infection progressed. WT group displayed a good control of MAP till day 12 post-infection, but a drop in the blood pressure at day 14 to a level similar to that observed in the *phox* KO group occurred. While WT mice restored blood pressure to normal levels, *phox* KO counterparts were unable to restore physiological MAP (Fig. 5B).

In order to verify the role of ^•^NO produced by NOS2 in the drop of blood pressure and in mortality, *phox* KO mice were treated with 1400W, a selective inhibitor of NOS2. Injections with 1400W were able to inhibit ^•^NO levels in the blood (data not shown) and to restore blood pressure levels (Fig. 5C). However, animals treated daily (not shown) or every 12 hours with 1400W displayed similar mortality rates to that of control mice (Fig. 5D). We treated the animals with a less selective NOS2 inhibitor (aminoguanidine) in the drinking water (1% w/v) from day 13 of infection and no effect was observed on the mortality of *phox* KO infected mice (data not shown). These treatments did not impact the control of parasite proliferation in either WT or *phox* KO animals, nor changed the outcome of the disease in WT mice (data not shown).

## Discussion

The involvement of ROS in host resistance against infectious diseases is well known [36], especially for bacterial and fungal infections. However, while some reports suggest the involvement of ROS in protozoa infections [1], [2], [3], [4], [5], others fail to find a major effect of these radicals in control of infections with *L. major* [6], *T. gondii* [37] and *Plasmodium* [9]. Importantly, chronic granulomatous disease patients are known to suffer from severe bacterial and fungal infections [38], but rarely from severe protozoa infections [39]. Interestingly, data from our laboratory suggests that infection with *T. cruzi* can induce a strong oxidative state in the host with production of ^•^NO, ROS and superoxide causing nitration of proteins in serum and target tissue [24] (and data not shown). ROS is known to be produced by macrophages following in vitro *T. cruzi* infection and to be one of the major oxidative agents on *T. cruzi*, reducing its viability dramatically [19], [21], [40]. In this study, we investigated the role of ROS on *T. cruzi* infection in vivo and surprisingly we found an important physiological effect of ROS, unrelated to the control of parasite.

In the present study, we found that animals deficient in gp91*^phox^* subunit of NADPH oxidase, a mouse model for chronic granulomatous disease [28], were able to efficiently control proliferation of Y strain of *T. cruzi*. Hence, parasitemia and parasite loads in spleen, liver and heart were similar in *phox* KO and WT mice. This result could suggest that ROS play a minor role in restriction of protozoal infection during *in vivo* infections. On the other hand, when carefully examined *in vitro*, the effects of ROS on parasite control can be appreciated, especially the effect of peroxynitrite. For example, macrophage-derived ROS and peroxynitrite were found to cause major oxidative burden on *T. cruzi*, reducing its viability dramatically [19], [21], [40]. Indeed, the virulence of different parasite strains can be predicted by the expression of some enzymes involved in the parasite anti-oxidant network such as TcTS, TXN, TcMPX, TcAPX and FeSOD-A [25]. The fact that macrophage-derived ROS were found to have little involvement in parasite control in *phox* KO mice may be related to other mechanisms of resistance operating *in vivo* such as compensatory ^•^NO production, p47GTPases expression [17], [18], CD8 T cells involvement [41] and alternative cellular sources of superoxide and peroxynitrite. Regarding this last point, we should indicate that normally, in activated macrophages, phagocyte-derived superoxide reacts with ^•^NO to yield peroxynitrite [21]; thus, in wild type animals superoxide from inflammatory cells plays a key role in ^•^NO-dependent cytotoxicity towards *T. cruzi* [20]. However, in the *phox* KO mice, the lack of macrophage-derived superoxide, increases the ^•^NO levels diffusing into the parasite, which in turn, inhibit the parasite mitochondrial respiration and secondarily enhance mitochondrial superoxide formation [25]. Overall, these processes lead to intramitochondrial formation of peroxynitrite and *T. cruzi* cytoxicity. Indeed, the exceeding available ^•^NO in *phox* KO could be responsible for parasite control, including the formation of peroxynitrite in parasite mitochondria [20] or by NOX4, recently found in macrophages [42]. Higher levels of ^•^NO found in sera from *phox* KO mice could not be attributed to higher expression of NOS2. This could be explained simply by the fact that ^•^NO is not reacting with superoxide to yield peroxinitrite in *phox* KO. Another possibility is raised by the fact that superoxide facilitates uncoupling of NOS and oxidation of tetrahidrobiopterin, therefore in its absence NOS would be more active and produce more ^•^NO [43].

In addition to their anti-infection role, ROS are involved in enhancing TLR signaling. Recently, it was demonstrated that ROS production is activated by TLR signaling through MyD88 and via the p38 MAPKinase cascade [44]. After their production is activated by TLR-dependent or independent pathways, ROS are able to enhance TLR4 expression on the cell surface [45] and to strength NF-κB activation [46]. The resistance to infection with *T. cruzi* is known to depend on appropriate MyD88 signaling [32] after stimulation of TLR2 and TLR9 [47], and TLR4 [48]. Although this function of ROS could result in improved immunity to *T. cruzi*, it seemed to have no critical role in our system. Our results show that *phox* KO mice exhibited no immune impairment, producing equivalent amounts of IFN-γ and TNF in response to infection and presenting similar histopathology (data not shown) to their WT partners.

Surprisingly, despite the ability of *phox* KO mice to restrict *T. cruzi* infection and mount an efficient immune response, they completely succumbed to infection by day 20 post-inoculation with *T. cruzi*. Further investigation showed that both WT and *phox* KO animals exhibited increased levels of NOx in sera from day 8 to 15 post-infection. However, the levels of nitrogen intermediates were higher in *phox* KO at day 15, coinciding with the initiation of mortality. ^•^NO is produced by three different isoforms of nitric oxide synthase (NOS1 or neuronal NOS, NOS2 or inducible and NOS3 or endothelial) and is known to play a pleiotropic role in host physiology [49], [50], [51], [52]. In addition to having potent anti-microbial properties, ^•^NO is involved in neurotransmission, gene expression and blood pressure regulation. For example, hyper-production of ^•^NO has severe consequences to the host, being the cause of hypotension during septic shock [49]. During *T. cruzi* infection, uncontrolled immune response has been proven to be deleterious to the host, as is the case of infection in the absence of IL10 [53], [54]. In *phox* KO animals excess ^•^NO was associated with peripheral blood pressure variations, not observed in WT controls and, more importantly, with early and permanent drop in central MAP. An important drop in MAP of WT animals was also observed, but this hypotension happened later than in *phox* KO mice and was transitory, lasting for no longer than 3 days. Although WT mice showed death rate of 40% starting on day 20 post-infection, we do not think this mortality is associated with the levels of ^•^NO since it starts after full recovery of blood pressure levels. From this study, we can conclude also that several factors might be involved in death associated with experimental T. cruzi infection. For example, the fact that treatment with 1400W prevented blood pressure drop in *phox* KO mice implicates NOS2. However, treatment was not able to prevent death. Inhibition of NOS using NOS inhibitors early in *T. cruzi* infection results in higher mortality due to infection [24], [55]. In contrast, treatment of *T. cruzi-*infected mice with NOS inhibitors in the chronic phase of the infection (Tulahuen strain) was not detrimental to the host's ability to control parasitism [35]. In addition, NOS2-deficient animals, in contrast to *ifn-γ* KO mice, can survive if treated with suboptimal doses of benzonidazole during peak of parasitemia even if the drug is withdrawn after parasite control [14]. We followed parasitemia in animals treated with NOS2 inhibitors after parasitemia was controlled and we did not observe recrudescence of parasite proliferation. These data suggest that ^•^NO may have an important role especially in the acute phase of the infection, in contrast to chronic phase when other IFN-γ-dependent mechanism controls the infection. The fact that NOS2 inhibition, although improving blood pressure, did not prevent mortality in our experiment could suggest that the cause of death may be multi-factorial possibly involving changes in hematological parameters (infection associated anemia and leucopenia) [56] or cardiac function [57], [58] and demands further investigation. However, data from shock models show that restoring blood pressures to normal levels may not rescue animals from death. The reason for this failure would be that the iNOS inhibition enhances the accumulation of activated leukocytes into vital organs, thus increasing tissue lesions. Also, inhibition of iNOS reduces the perfusion of the organs [59], [60], [61], [62], [63].

Another very interesting side of ROS actions started to be depicted recently. ROS have been shown to regulate vasoactive properties of ^•^NO. Nitric oxide is known to react with the heme group of guanylate cyclase activating the production of cGMP that promotes vasodilation [64]. Accordingly, some inhibitors of guanylate cyclase, such as methylene blue, induce ROS production. In addition, ROS derived from endothelial NAPDH oxidase containing gp91*^phox^* is a potent vasoconstrictor because it scavenges ^•^NO before ^•^NO activates guanilate cyclase [29], [30]. Hence, one unifying hypothesis to explain an important part of our observations is that *T. cruzi* infection stimulates a strong production of ^•^NO and *phox* KO animals cannot produce ROS in order to counteract the systemic effect of ^•^NO. Coherently, despite elevated levels of NOx detected in sera of *phox* KO animals, they expressed similar levels of NOS2 by real-time RT-PCR in the spleen. This fact and the finding that IFN-γ and TNF are not increased in *phox* KO animals suggest that it is not likely that elevated NOx in serum is due to augmented production, but may be related to impaired ROS production and its role in scavenging ^•^NO. We propose that the reaction of ROS and ^•^NO to generate peroxynitrite, in addition to strengthening the killing effects of ^•^NO by augmenting its oxidative properties [21], has an important role in regulating ^•^NO signaling and its systemic effects during *T. cruzi* infection.
